# Motor Resonance Flexibility to Emotion-Enriched Context in Parkinson's Disease Patients

**DOI:** 10.1155/2022/6487419

**Published:** 2022-12-30

**Authors:** Giovanna Lagravinese, Ambra Bisio, Marco Bove, Alessandro Botta, Gaia Bonassi, Roberta Marchese, Piero Ruggeri, Elisa Pelosin, Laura Avanzino

**Affiliations:** ^1^Department of Neuroscience, Rehabilitation, Ophthalmology, Genetics, Maternal and Child Health, University of Genoa, Genoa 16132, Italy; ^2^IRCCS, Ospedale Policlinico San Martino, Genoa 16132, Italy; ^3^Department of Experimental Medicine, Section of Human Physiology, University of Genoa, Genoa 16132, Italy; ^4^S.C. Medicina Fisica e Riabilitazione Ospedaliera, ASL4, Azienda Sanitaria Locale, Chiavari, Italy

## Abstract

In healthy people, motor resonance mechanisms are flexible to negative emotional contextual clues with greater motor resonance during the observation of a reach to grasp movement performed in an environment eliciting disgust. The link between emotion and motor control has become an interesting topic in Parkinson's disease (PD). Here, we aimed to study the response of the mirror neuron system, specifically motor resonance, to an emotion-enriched context in people with PD. Corticospinal excitability was recorded in a total of 44 participants, divided into two groups (23 PD patients and 21 healthy subjects). We recorded motor-evoked potentials from a muscle involved in the grasping movement while participants were watching the same reach-to-grasp movement embedded in surrounds with negative emotional valence, but different levels of arousal: sadness (low arousal) and disgust (high arousal). Basic motor resonance mechanisms were less efficient in PD than controls. Responsiveness to emotional contextual clues eliciting sadness was similar between PD and controls, whereas responsiveness to emotional contextual clues eliciting disgust was impaired in PD patients. Our findings show reduced motor resonance flexibility to the disgusting context, supporting the hypothesis that PD patients may have a deficit in “translating” an aversive motivational state into a physiologic response. The amygdala, which is implicated in the appraisal of fearful stimuli and response to threatening situations, might be implicated in this process.

## 1. Introduction

It is known that when we understand and predict other people's actions we activate our “mirror neuron system” [1–3]. Observation of others' actions evokes a subliminal motor response (i.e., motor resonance), which reflects the motor program encoding the observed action. Transcranial magnetic-stimulation (TMS) studies have shown a corticospinal excitability facilitation (larger amplitude of motor evoked potentials, MEPs) during action observation, suggesting a role for the primary motor area in motor resonance .

In a previous research, we looked at how watching a movement carried out in a negative emotional setting affected motor resonance [4]. We concentrated on negative emotions (sadness and disgust) at various levels of arousal [4]. Our findings suggested that the emotional context in which a movement occurs increases motor resonance in a combination of negative valence/high arousal contexts, as evidenced by greater motor resonance (i.e., larger amplitude of MEPs) during the observation of the movement in the context eliciting disgust compared with the others [4]. Even if we decided to focus our attention on negative valenced emotional contexts, it is worthy to report some studies investigating the impact of others emotional stimuli on motor excitability. Using different stimuli (pictures, body postures, and facial expressions) and different emotions (happiness, fear, and neutral), Borgomaneri et al. [5–7] showed different effects of the stimuli on motor excitability, particularly at early timing, that is, 150 ms after stimulus presentation. While negative pictures increased motor excitability specifically for the perception of negative pictures [5], body postures evoked a reduction in MEPs amplitude both for happy and fear emotions [6]. On the other hand, the opposite result was obtained when facial expressions were used, with an increase of corticospinal excitability for happy and fearful emotional faces [7]. These results indicate that emotion perception is closely linked to action systems and that not only the level of arousal is a key factor for changes in motor excitability, but different stimuli might involve motor resonance mechanisms rather than emotion-related motor modulations.

Here, we aim to study motor resonance flexibility to emotion-enriched contexts in Parkinson's disease (PD). Although PD is considered a movement disorder, as diagnosis is based on cardinal motor signs and symptoms [8], affective disorders (particularly depression and anxiety) are common and disabling [9]. An altered motor control concerning facial expressions, upper limb fine performance, and gait has been observed in PD in relation to emotional processing [10–15], and particularly to processing of negative emotions [10–15]. Indeed, it has been demonstrated that both spontaneous and posed facial expressions are altered and diminished in PD patients [10, 11]. Emerging evidence showed that emotional disturbances arising from affective disorders, such as anxiety and depression, could contribute to gait disorders in some people with PD. A recent study elegantly showed that stress-evoking emotional stimuli increase deficits in fine motor control in PD [15].

In accordance with the mirror neuron theory, which postulates the overlap of neural mechanisms mediating action production and action understanding [16]; here, we aimed to explore whether motor resonance flexibility to emotion-enriched context is altered in patients with PD.

We investigated how different levels of arousal during the observation of a similar reach-to-grasp hand movement in a scenario with negative emotional valence affected motor resonance in PD patients. Based on the above reported premises we expected that the mirror system would be less responsive to emotional stimuli in patients with PD.

## 2. Materials and Methods

### 2.1. Participants

A total of 23 PD patients and 21 healthy age-matched subjects (HS) were recruited at the Department of Neuroscience, University of Genoa, Genoa, Italy. All participants were right-handed, according to Right Edinburgh Handedness Inventory. Patients were enrolled if they met the following inclusion criteria: (i) diagnosis of idiopathic PD (according to the United Kingdom Parkinson's Disease Society Brain Bank criteria) and (ii) Hoehn and Yahr stage ≤3. Participants were excluded in the presence of (i) Montreal Cognitive Assessment (MoCA) score <24, (ii) history of neurologic disorders (except PD), (iii) deep brain stimulation implant, and (iv) other contraindications for TMS. HS were enrolled if they met the following inclusion criteria: (i) MoCA score >24 and (ii) no contra-indications for TMS. All PD patients were under treatment with dopaminergic therapy, and the experiment took place during the “ON” state. Before taking part, all participants provided signed informed consent. The University of Genoa's ethics committee accepted the experimental protocol (141/12), and it was carried out in accordance with all applicable laws and conventions worldwide.

### 2.2. Clinical Assessment

Prior to completing the TMS protocol, PD participants completed a set of questionnaires and examinations to assess motor symptoms (MDS-UPDRS III, Hoehn and Yahr stage), the presence of affective (Beck Depression Inventory [BDI-2] and Beck Anxiety Inventory [BAI]) and cognitive symptoms (MoCA). Dopamine equivalent dose was calculated by using previously established guidelines. Finally, affective theory of mind ability was assessed using the Reading the Mind in the Eyes test [17].

### 2.3. Experimental Paradigm

**Figure 1:**
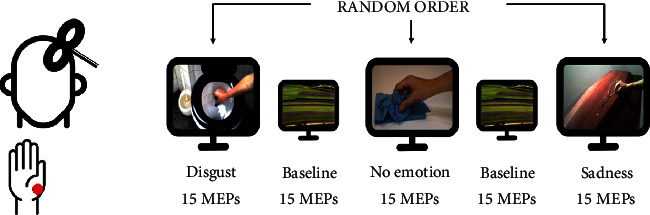
Experimental paradigm. Corticospinal excitability of the left primary motor cortex was evaluated while subjects were watching a reach-to-grasp movement embedded in two emotional contexts (disgust and sadness) and in a no-emotion context. Motor-evoked potential (MEPs) were recorded from abductor pollicis brevis (APB) muscle. A video showing a landscape was used as wash out (baseline).

The experimental paradigm is depicted in Figure 1. Subjects were instructed to carefully view videos or images displayed on a 19″ screen that was 60 cm away from them while they were seated in a comfortable chair. TMS was used to measure the cortical excitability of the left primary motor cortex (M1) as participants watched videos and images. Participants watched a black screen for 3 seconds before the next video was displayed. Each video or image lasted 5 seconds. Each video was shown 15 times in a row, followed by a baseline block of 15 landscape images. We used three videos showing a right hand reaching different objects. Two videos featured a hand reaching for various items in emotionally charged situations. They specifically portrayed a hand grabbing a rosary put on a coffin to evoke sadness (sadness video), and a hand grasping extremely soiled toilet paper to evoke disgust (disgust video). In a third video, the identical action was depicted in a neutral setting with a hand holding a napkin on a table (no-emotion video). A precision-grip movement that only uses the abductor pollicis brevis (APB) muscle concluded each movement. The three video blocks were shown in a random order. The right APB cortical area's excitability was investigated. The Self-Assessment Manikin (SAM) scale [18] was used to ask participants to rate the level of emotional valence and arousal evoked by the movies at the conclusion of each experimental condition. In our scoring system, a score of 5 indicates a high rating on each dimension (i.e.., pleasant, high arousal), and a score of 0 indicates a low rating on each dimension (i.e., unpleasant, low arousal).

### 2.4. Transcranial Magnetic-Stimulation

Focal TMS was applied on left M1 with a single Magstim 2002 magnetic stimulator (Magstim Company, Whitland, UK) connected with a figure-of-eight coil (wing diameter: 70 mm). The coil was placed tangentially to the scalp with the handle pointing backward and laterally at 45° to the sagittal plane inducing a postero-anterior current in the brain. We determined the optimal position for activation of the right APB muscle by moving the coil in 0.5 cm steps around the presumed motor hand area. At the beginning of the experimental condition, the stimulus level required to elicit a mean MEP with an amplitude of roughly 0.8–1 mV peak-to-peak was determined for each participant. This intensity was employed throughout the entire experiment. The delivery of the magnetic stimulation and the presentation of the visual stimulus were timed using specialized MATLAB software. Specifically, the magnetic stimulus was randomly delivered 150 ms before or 150 ms after the contact between the hand and the object. The TMS stimulation was randomly administered in the baseline condition while the landscape was visible on the screen. A total of 15 MEPs from the target muscle were recorded during each experimental session.

### 2.5. Electromyographic Recording

Silver disc surface electrodes were used to record electromyographic (EMG) activity. These electrodes were placed over the right hand's APB muscle belly and related tendon. A ground electrode was placed at the elbow. EMG was digitized, amplified, and filtered (20–1 kHz) with a 1902 isolated pre-amplifier controlled by the Power 1401 acquisition interface (Cambridge Electronic Design Limited, Cambridge, UK), and then recorded on a computer for offline data processing. A recording epoch lasted 400 ms, of which 100 ms occurred prior to the TMS. Throughout the entire experiment, participants were encouraged to always maintain their hand relaxed. To prevent muscle activity from influencing action observation trials, we carefully controlled the EMG activity in real-time. All trials had muscular activity that was less than 10 *μ*V and similar to that of the muscle at rest (*p* > 0.1).

### 2.6. Data Analysis

For neurophysiological data, measurements of MEPs were made on single trials. The amplitude of MEPs recorded from right ABP was evaluated by taking the peak-to-peak difference in the raw EMG signals. Mean values of MEPs amplitude were calculated for each subject, in each experimental condition. A “Baseline” condition was established using the average values of all MEPs collected throughout the landscape image presentation. For the emotional conditions (sadness and disgust) we also calculated a “motor resonance flexibility index” (MRFI), reporting the amount of influence of emotional context on motor resonance with the following formula:

(1) $$\begin{array}{c} \text{MRFI}=\left(\text{MEPs emotion}-\text{MEPs no}-\text{emotion}/\text{MEPs baseline}\right)\times 100. \end{array}$$

Mean and standard deviations were computed for SAM scale.

### 2.7. Statistical Analysis

Chi-square test was applied to assess gender differences between groups (HS and PD). Differences between groups for age, that was normally distributed, were assessed by a one-way analysis of variance (ANOVA).

MEPs data were subjected to a repeated measures (RM)-ANOVA with type of video (disgust, sadness, no-emotion, and baseline) as a within-subjects factor and group as a between-subjects factor, to assess variations in M1 excitability. An RM-ANOVA was conducted for MRFI with group as the between-subjects factor and emotional video (disgust and sadness) as the within-subjects factor. For the SAM scale, valence and arousal ratings on disgust and sadness videos were analysed with two separate Mann–Whitney *U* test since data were not normally distributed. Furthermore, to compare valence and arousal ratings within PD and HS groups, two Wilcoxon tests were performed. Finally, to explore a possible association between psychological variables (depression, assessed by BDI-2; anxiety, assessed by BAI; and theory of mind ability, assessed by RMET) with the MRFI, we performed correlation analysis for PD patients. In case of significant correlation, Bonferroni correction was applied (0.05/3 = 0.016). We also explored a possible association between PD disease severity (MDS-UPDRS III, Hoehn and Yahr stage) with the MRFI, by performing a correlation analysis; in case of significant correlation, Bonferroni correction was applied (0.05/2 = 0.025). The statistical analyses were performed with SPSS (Statistical Package for the Social Sciences, SPSS 20, Chicago, IL, USA).

## 3. Results

### 3.1. Participant Characteristics

The statistics for demographic, clinical data, and neuropsychological tests are reported in Tables 1 and 2.

### 3.2. Motor Resonance Flexibility

**Figure 2:**
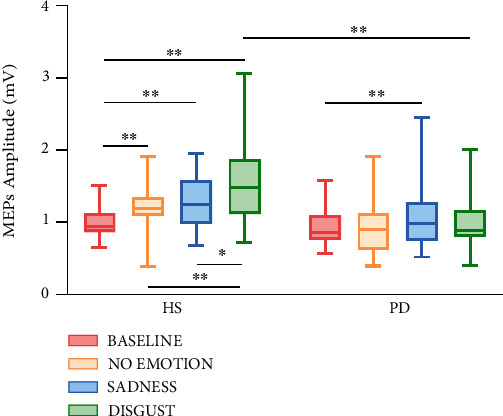
Corticospinal excitability of the left primary motor cortex evaluated during the observation of different types of videos in two different groups of subjects (patients with Parkinson's disease, PD; healthy subjects, HS). *Y*-axis represents the motor evoked potentials (MEPs) amplitude (in mV). Box plots report the interquartile range and the median values. Asterisks indicate the level of significance (∗*p* < 0.05 and ∗∗*p* < 0.01).

Data on motor resonance flexibility are reported in Figure 2. RM-ANOVA of raw MEPs data displayed a main effect of type of video ((*F*(3,126) = 9.05, *p* < 0.0001) and a significant type of video × group interaction (*F*(3,126) = 6.075, *p* = 0.001).

MEPs recorded at baseline were considerably lower than MEPs recorded while individuals watched all the videos showing the grasping movement, according to post-hoc analysis (baseline vs. no-emotion, *p* = 0.007; baseline vs. sadness, *p* < 0.0001; and baseline vs. disgust, *p* < 0.0001). Post-hoc analysis of the interaction showed that MEPs recorded at the baseline were considerably lower than MEPs recorded while simply viewing all grasping actions only in HS (baseline vs. no-emotion, *p* = 0.004; baseline vs. sadness, *p* < 0.0001; and baseline vs. disgust, *p* < 0.0001). Furthermore, in HS, MEPs recorded during the viewing of the disgust video were considerably greater than MEPs recorded during the viewing of the no-emotion and sadness videos (*p* < 0.0001 and *p* = 0.016, respectively). For PD, MEPs collected in the baseline were significantly lower only with respect to MEPs collected in the sadness condition (*p* = 0.020). Finally, MEPs were considerably higher in the disgust condition in HS compared with the same video in PD (*p* = 0.002).

**Figure 3:**
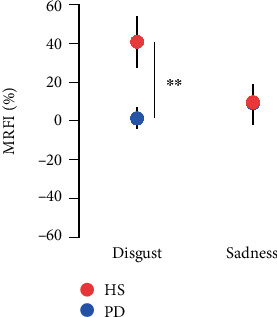
Motor resonance flexibility index (MRFI) which represents the amount of influence of emotional context on motor resonance calculated with the following formula: MRFI = (MEPs emotion − MEPs no-emotion/MEPs baseline) × 100 in PD patients and healthy subjects (HS). Vertical bars indicate SE.

RM-ANOVA on motor flexibility index displayed a significant emotional video × group interaction (*F*(1, 42) = 6.52, *p* = 0.014; Figure 3). Post-hoc analysis showed that the effect of disgust on cortical excitability was significantly higher in HS than in PD (*p* = 0.005). Any difference emerged between HS and PD (*p* = 0.96) for sadness video.

### 3.3. SAM Scores

Statistical analysis showed no differences between groups for valence nor for arousal (*p* > 0.05; Table 3). Comparison within groups revealed that for arousal, both PD and HS gave a lower arousal score for sadness video with respect to disgust video (*p* < 0.005).

Regarding valence, only PD perceived the disgust video more unpleasant than the sadness one (*p* = 0.01).

### 3.4. Correlation Analysis

No correlation emerged between neuropsychological characteristics and disease severity scores and the MRFI in PD (*p* always > 0.05).

## 4. Discussion

In this study, we tested motor resonance while patients affected by PD were observing videos of a grasping movement embedded in different emotional contexts.

The main findings of the present study were the following: (i) basic motor resonance mechanisms were less efficient in PD than controls; (ii) responsiveness to emotional contextual clues eliciting sadness was similar between PD and controls; and (iii) responsiveness to emotional contextual clues eliciting disgust was impaired in patients with PD.

A first consideration regards motor resonance in PD. Motor resonance is sub-served by a frontoparietal mirror neuron system, identified in humans through neuroimaging and electrophysiological studies, as part of a broader action observation network [19]. The basal ganglia are likely to be involved in this network, based on neuroimaging evidence from healthy individuals [20] and subthalamic nucleus recordings in PD [21]. Thus, reduced motor resonance might be expected in PD. However, data in the literature are controversial. Tremblay et al. showed reduced modulation of MEPs during action observation in people with PD respect to controls [22]. In contrast, Bek et al. [23] found intact motor resonance in people with PD. Similarly, we showed that it is possible to induce a postural contagion (chameleon-like mimicry effect) throughout the observation of stimuli displaying human imbalance in patients with PD [24]. Furthermore, action observation training can be useful to improve movement and functional independence [25, 26] and gait [26] in people with PD. Our results show impaired motor resonance to action observation in PD, but also show that when the movement is embedded in an emotional context eliciting sadness, motor resonance mechanisms are comparable to those of controls. Sadness is a commonly experienced emotion, impacting body and mind [27]. Although the average BDI-2 score of PD group (*x̅* = 8.77, SD = 4.35) fell below the recommended clinical cut-off (approximate range 13–14) for depression in PD [28], living with a chronic disease can promote the feeling of sadness, probably not detected by the BDI-2 test because not so impacting on quality of life. Brain mechanisms for action understanding have been proven to rely on matching the observed actions into the viewer's motor system. As an example, the observation of unusual pathological actions differently modulates the viewer's motor system, depending on knowledge and visual expertise. [29]. Following this line of reasoning, we can speculate that the preserved motor resonance for reaching movements embedded in the “sadness” context observed in PD would have been driven by PD patients' expertise in sadness feeling.

Related to disgust, this has been categorized as a negative, high arousal emotion, differently from sadness, which represents a negative, low arousal emotion. In our earlier research, we proposed that in healthy individuals, the increase in motor resonance elicited by disgust could be viewed as a motor system response to a condition that is preferable to flee or avoid. This outcome supported the notion that arousal is a crucial first stage in both animals and humans' activation of defence behaviour (for a review see [30]). Interestingly, the link between disgust and a protective behaviour has been observed also on tongue M1 cortico-hypoglossal excitability (tMEP), with a reduction of tMEPs amplitude during the observation of pictures related to gustatory disgust and revulsion (i.e., rotten food), indicating that disgust is able to influence the motor system in order to avoid ingestion of contaminants [31]. Moreover, also disgust related to moral indignation seems to influence tM1 excitability: in participants who disapproved some vignettes which described moral violations, a reduction of tM1 excitability was observed [32], supporting the hypothesis that morality might have originated from the more primitive experience of oral distaste.

Our finding related to reduced motor system's reaction to unpleasant high-arousal stimuli in PD is in accordance with previous findings [33, 34]. PD patients have been shown to exhibit smaller startle eyeblink response than controls while viewing unpleasant, aversive pictures, evoking both fear and disgust. The enhancement of startle, normally expressed by controls when viewing unpleasant, aversive picture has been interpreted as a protective withdrawal reflex, primed during unpleasant emotional states and inhibited during pleasant emotional states [35]. Interestingly, in a series of works, it has been shown that reduced startle eyeblink in PD is mediated by arousal level of the pictures, similarly to what happens to motor resonance flexibility presented here. In the control group, arousal level modulated the startle reflex, whereas this did not happen in PD patients, showing similar startle eyeblink magnitudes to both low arousal and high arousal pictures. In this scenario, reduced motor resonance flexibility to disgust context supports the hypothesis that PD patients may have a deficit in “translating” an aversive motivational state into a physiologic response.

A point to be discussed regards the possible network involved in dysfunctioning motor resonance flexibility to emotional contextual clues in PD. One structure that may play a key role is the amygdala, which has consistently been implicated in response to threatening situations [36]. An amygdala-based translational defect may be hypothesized; whereby, the results of cognitive appraisal are not appropriately transcoded into somato-motor-arousal responses normally associated with an aversive motivational state. This may arise from faulty dopaminergic gating of the amygdala, resulting in “inhibition” of the amygdala [37]. It has been proposed that, by acting on specific interneurons of the basolateral and central amygdala, dopamine acts as a switch between cortically controlled and disinhibited states of the amygdala. In PD, disease-related dopaminergic depletion, might minimize the extent to which the amygdala becomes “disinhibited” during aversive situations, resulting in diminished threat-related reactivity.

Finally, our results suggest that motor resonance flexibility in PD was not influenced by differences in emotion perception between PD and HS or in cognitive and affective status of PD patients. Indeed, although there were differences in self-perceived evaluation of the emotional context between PD and HS, these differences did not influence motor resonance flexibility, as valence and arousal ratings did not correlate with motor resonance flexibility to emotional contexts. At the same time, no correlation was found between affective and cognitive and disease severity-related measures and motor resonance flexibility.

## 5. Limitations and Conclusions

Some limitations of the study deserve discussion. The first is the limited sample size, which limited us in analysing whether motor resonance flexibility was influenced by PD phenotype. Second, future studies should directly address the network responsible for diminished motor resonance flexibility in PD.

To conclude, our findings show that reduced motor resonance flexibility to the disgusting context, supporting the hypothesis that PD patients may have a deficit in “translating” an aversive motivational state into a physiologic response. This piece of information enriches the literature on the link between emotional processing and motor control in PD, which represents a typical basal ganglia disease. Finally, the mirror neuron system is an integral component of the social brain and is likely to play a critical functional role in the spontaneous or automatic processing of social cognition. Given the fact that motor resonance implies one's capacity to embody a representation of others' actions, and it seems to contribute to several complex and crucial social skills, the impaired motor resonance flexibility exhibited by PD may theoretically have an impact on social cognition and patients' quality of life.

## Figures and Tables

**Table 1:** Demographic and clinical characteristics.

	PD	HS	*p*-Value
Number of subjects	(14 male and 9 female)	(9 male and 12 female)	*χ*2 = 1.42, *p* = 0.23
	Mean ± SD	Mean ± SD	
Age (years)	70.39 ± 4.5	69.11 ± 4.83	*p* = 0.06
Education (years)	11.65 ± 3.37	12.66 ± 4.92	*p* = 0.50
Disease duration (years)	7.18 ± 3.8	—	—
Hohen and Yahr (stage)	1.92 ± 0.69	—	—
LEDD (mg)	863.28 ± 296.27	—	—
UPDRS part III (score)	22.84 ± 13	—	—

PD, Parkinson's disease; HS, healthy subjects; UPDRS, Motor Section of the Movement Disorder Society Unified Parkinson's Disease Rating Scale; LEED, levodopa equivalent daily dose.

**Table 2:** PD Neuropsychological characteristics.

Number of subjects	23
	Mean ± SD
MoCA (score)	27 ± 2.36
BDI-2 (score)	8.77 ± 4.35
BAI (score)	6.86 ± 5.75
RMET (% correct responses)	56.67 ± 5.8

PD, Parkinson's disease; MoCA, Montreal Cognitive Assessment; BDI-2, Beck Depression Inventory 2; BAI, Beck Anxiety Inventory; RMET, reading the mind in the eyes test.

**Table 3:** Emotional valence and emotional arousal (mean ± SD).

	HS		PD		*p*-Value
	Sadness	Disgust	Sadness	Disgust	
Valence	1.39 ± 0.65	1.29 ± 0.7	1.63 ± 0.71	1.22 ± 0.42	>0.05
Arousal	2.28 ± 0.92	3.30 ± 0.97	2.52 ± 0.99	3.26 ± 1.21	>0.05

PD, Parkinson's disease; HS, healthy subjects.

## Data Availability

Data supporting this research article are available from the corresponding author or first author on reasonable request.
